# Safety and Efficacy of Degradable Starch Microspheres Transcatheter Arterial Chemoembolization (DSM-TACE) in the Downstaging of Intermediate-Stage Hepatocellular Carcinoma (HCC) in Patients With a Child-Pugh Score of 8-9

**DOI:** 10.3389/fphar.2021.634087

**Published:** 2021-04-08

**Authors:** Roberto Minici, Michele Ammendola, Francesco Manti, Maria Anna Siciliano, Marco Minici, Iman Komaei, Giuseppe Currò, Domenico Laganà

**Affiliations:** ^1^ Radiology Unit, Department of Experimental and Clinical Medicine, Magna Graecia University, Catanzaro, Italy; ^2^ Digestive Surgery Unit, Science of Health Department, Magna Graecia University, Catanzaro, Italy; ^3^ Medical Oncology Unit, Department of Experimental and Clinical Medicine, Magna Graecia University, Catanzaro, Italy; ^4^ National Research Council (Cnr), Institute for High Performance Computing and Networking (ICAR), Rende, Italy; ^5^ Department of Human Pathology of Adult and Evolutive Age, University of Messina, Messina, Italy; ^6^ General Surgery Unit, Science of Health Department, Magna Graecia University, Catanzaro, Italy

**Keywords:** hepatocellular carcinoma, downstaging, transcatheter arterial chemoembolization, degradable starch microsphere, transarterial, doxorubicin, stromal microenvironment, tumoral angiogenesis

## Abstract

According to the EASL Guidelines for the management of hepatocellular carcinoma, transcatheter arterial chemoembolization is the first-line treatment recommended for intermediate-stage HCC. Furthermore, it is widely accepted that patients beyond the Milan criteria can be considered for a liver transplant after successful downstaging to within the Milan criteria. Response to downstaging treatments significantly influences not just drop-outs, but also the rate of post-transplantation tumor recurrences. TACE with degradable starch microspheres represents an alternative to conventional TACE with lipiodol and TACE with drug-eluting beads, and it leads to transient arterial occlusion allowing lower activation of hypoxia-inducible factors and less release of vascular endothelial growth factor, a promoter of neoangiogenesis, tumor proliferation, and metastatic growth. In patients with intermediate-stage HCC and a Child-Pugh score of 8 or 9, life expectancy may be dominated by cirrhotic liver dysfunction, rather than by the tumor progression itself; hence, locoregional treatments might also be detrimental, precipitating liver dysfunction to an extent that survival is shortened rather than prolonged. Data on tolerability, toxicity, and effectiveness of DSM-TACE are limited but encouraging. Between January 2015 and October 2020, 50 consecutive patients with intermediate-stage hepatocellular carcinoma and a Child-Pugh score of 8/9, who had undergone DSM-TACE as the first-line treatment, were eligible for the study. A total of 142 DSM-TACEs were performed, with a mean number of 2.84 procedures per patient. The mean time-to-downstaging was 19.2 months, with six patients successfully downstaged. OS was about 100% at six months, 81.8% at 12 months, and 50% at 24 months. Twenty-two patients experienced adverse events after chemoembolization. The median OS and safety of DSM-TACE in this study are comparable with other published investigations in this field. Furthermore, 12% of patients were successfully downstaged. Hence, the results of the current investigation demonstrate that DSM-TACE is effective and safe in intermediate-stage HCC, achieving an interesting downstaging rate. Such data were observed in the population subset with a Child-Pugh score of 8 or 9, in which life expectancy may be determined by cirrhotic liver dysfunction, so the achievement of a balance between the safety and efficacy profile of the TACE treatment is crucial.

## Introduction

Liver cancer has the seventh highest number of new cases and is the third leading cause of cancer-related death worldwide, with 841,080 new cases and 781,631 deaths per year (Bray et al., 2018). According to the EASL Guideline for the management of hepatocellular carcinoma (Galle et al., 2018), TACE is the first-line therapy recommended for intermediate-stage hepatocellular carcinoma or stage B disease, classified according to the Barcelona-Clinic Liver Cancer staging system (Llovet et al., 1999; Forner et al., 2010). The efficacy of TACE has been investigated in a few randomized trials compared with best supportive care and in a cumulative meta-analysis (Llovet and Bruix, 2003). The benefit of TACE in improving overall survival was demonstrated for patients in the Child-Pugh A stage, with early or intermediate BCLC disease, who had a small tumor burden but were unfit for surgery and for ablative therapies. In this population, it is possible to assume a median OS of 30–45 months (Golfieri et al., 2014; Burrel and Reig, 2012). A median OS inferior to 20 months has been reported in real-life cohorts when patients with no proven benefit are treated including those in the Child-Pugh B stage or those with deteriorating liver function under TACE (Kirstein et al., 2017). According to the EASL Guideline for the management of hepatocellular carcinoma (Galle et al., 2018), patients with intermediate-stage HCC and Child-Pugh A-B liver function may have a dismal outcome without transplantation if refractory ascites and events such as spontaneous bacterial peritonitis, hyponatremia, or recurrent encephalopathy occur. In such a cohort, a liver transplant is reckless and if HCC surpasses the agreed criteria, the patient must be classified as BCLC-D. Interventional studies usually exclude this population subset because of its high short-term mortality. Hence, a broad variety of patients are included in the intermediate-stage HCC definition, triggering controversies to further stratify the BCLC-B category (Bolondi et al., 2012). Furthermore, it is widely accepted that patients outside the Milan criteria can be considered for liver transplant after successful downstaging to within the Milan criteria, within specified protocols (Galle et al., 2018). Response to downstage therapies has a major effect not just on drop-outs, but also on the rate of post-transplantation tumor recurrences (Cucchetti et al., 2011; Yao et al., 2015). Interestingly, the efficacy of downstage therapies is correlated with the presence of histological markers of good prognosis in the treated HCC, (i.e. the absence of microvascular invasion and satellites, low-grade tumor), similar to patients receiving LT within the Milan criteria at presentation. Thus, the response to downstaging has an important role in predicting tumor aggressiveness (Mazzaferro et al., 2011; Yao et al., 2015). Among locoregional therapies to downstage HCC, TACE is widely used and investigated in clinical practice (Parikh et al., 2015; Bryce and Tsochatzis, 2017; Affonso et al., 2019) and in small case-series regarding TACE with degradable starch microspheres (Orlacchio et al., 2015). DSM-TACE represents an option with respect to conventional TACE with lipiodol or TACE with drug-eluting beads (DEB-TACE) (Gross and Albrecht, 2020). Starch microspheres lead to transient arterial occlusion of a maximum of 50 min *in vivo* before they are degraded by serum alpha-amylase (Aronsen et al., 1979), achieving an increase in the intratumoral concentration of the cytostatic agent (Ebert et al., 2013). Temporary occlusion decreases the stimulation of hypoxia-inducible factors and the release of vascular endothelial growth factor, a promoter of neoangiogenesis, tumor proliferation, and metastatic growth (Li et al., 2004), making DSM-TACE a powerful tool among therapeutic strategies to control cancer, targeting the tumor stromal environment in combination with classic chemotherapeutic agents. However, there is a paucity of data on the tolerability, toxicity, and effectiveness of DSM-TACE (Niessen et al., 2014), and further clinical investigations should be encouraged. In patients with intermediate-stage HCC and a Child-Pugh score of 8 or 9, life expectation is influenced by cirrhotic liver dysfunction, rather than by tumor growth itself (Bolondi et al., 2012). Hence, locoregional treatments could downstage disease to access LT, that is the only approach with curative intent. On the other side, locoregional treatments might also be detrimental, precipitating liver dysfunction to the extent that survival is shortened rather than prolonged. Technically, there could be certain patients in this population subset, without jaundice and with only moderate ascites, who may be candidates for super-selective DSM-TACE, a tool that can face the challenge of downstaging disease within the Milan criteria, keeping an acceptable safety profile. These patients, if transplanted, may achieve good survival, which exceeds 50% at five years (Mazzaferro et al., 2009). Considering what was previously written, this study aims to evaluate the safety and efficacy of DSM-TACE in the downstaging of intermediate-stage hepatocellular carcinoma in patients with a Child-Pugh score of 8 or 9.

## Materials and Methods

### Study Design

Institutional Review Board approval and informed written consent from each patient was obtained. This study is a single-center, retrospective analysis of prospectively collected data of consecutive patients with intermediate-stage hepatocellular carcinoma and a Child-Pugh score of 8/9, who had undergone, from January 2015 to October 2020, DSM-TACE as the first-line treatment.

Inclusion criteria were: I) intermediate-stage (B)—according to the Barcelona-Clínic Liver Cancer staging system (Llovet et al., 1999; Forner et al., 2010)—hepatocellular carcinoma, diagnosed with histological assessment or non-invasive imaging-based criteria used by the European Association for the Study of the Liver (Galle et al., 2018); II) Child-Pugh stage B with a score of 8 or 9; III) age between 18 and 75 years; IV) no previous treatment for HCC; V) Eastern Cooperative Oncology Group performance status (Oken et al., 1982) grade 0; and VI) evaluation by a multidisciplinary team of a hepatologist, oncologist, liver surgeon, and interventional radiologist. The exclusion criteria were: I) missed radiological evaluations at follow-up; II) serum creatinine levels >2.0 mg/dl; III) platelet count <50000/μL and/or international normalized ratio >1.5; IV) serum bilirubin level ≥3 mg/dl; and V) doxorubicin administration contraindications.

### Intervention

At baseline condition, at least three weeks before the first treatment, all patients underwent a clinical, biochemical, and imaging examination. Imaging evaluation was performed with contrast-enhanced computed tomography and/or gadolinium-enhanced magnetic resonance imaging, using a multiphase liver imaging protocol.

DSM-TACE was performed according to the evaluation made by a multidisciplinary team. DSM-TACE was carried out in a dedicated angiography suite, monitoring vital signs during anesthesia, by the same experienced interventional radiologists (30 and 2 years of experience, respectively). All patients were pre-medicated with a proton-pump inhibitor (omeprazole 40 mg i.v.), a prokinetic drug (metoclopramide 10 mg i.v.), and an analgesic drug (ketorolac-tromethamine 20 mg i.v.); if requested, conscious sedation was performed during the procedure. The treatment was performed through a femoral or radial approach, with a Seldinger needle, by using a 5-Fr arterial introducer sheath (Terumo, Tokyo, Japan). The selective celiac trunk catheterization and the cannulation of the common hepatic artery were performed with a 5-Fr diagnostic catheter (Cobra, Simmons; Terumo). The appropriate anatomy of the hepatic artery and any possible branches related to non-target structures and any possible arteriovenous fistulae were identified through a hepatic artery angiography. After diagnostic angiography, a selective lobar catheterization was performed with a coaxial technique, placing a 2.7-Fr microcatheter (Progreat; Terumo) in the right or left hepatic artery that was feeding the involved lobe. A selective lobar angiography was then performed to confirm the correct position of the microcatheter, to identify non-hepatic arteries, and limit any possible extrahepatic diffusion of the microspheres. In particular, the identification of the cystic artery was recommended to ensure that the catheter tip would bypass this anatomical point to avoid non-target embolization. A super-selective (segmental or sub-segmental) approach was obtained using the aforementioned microcatheter. DSMs were mixed with non-ionic iodinated contrast medium: 6 ml of nonionic iodinated contrast was used per 4 ml of DSMs before injection. Doxorubicin at a dose of 50 mg was diluted in 5 ml of normal saline. No dose adjustment was made for bilirubin concentration or body surface area. An appropriate suspension of DSMs, contrast medium, and doxorubicin was obtained before delivery. The mixture in the syringes was constantly shaken to avoid sedimentation and disaggregation of the microspheres, then slowly injected under fluoroscopic guidance at the proper site, until stasis was observed. Stasis was defined as the absence of antegrade flow within a vessel such that contrast filling of the target vessel persisted, without washout, five cardiac beats after the injection of contrast (Brown et al., 2016). When stasis had been reached, a mixture of starch microspheres (4 ml) with contrast medium (6 ml) was slowly injected until a complete embolization was obtained.

All patients underwent physical examination, laboratory tests, and imaging follow-up at one month after each treatment and every three months thereafter if no additional treatment was required. For each patient, the imaging modality (an abdominal contrast-enhanced CT or MRI examination) remained the same throughout the entire study period.

DSM-TACE treatments were repeated on-demand upon the demonstration of a viable tumor (absence of CR) in patients who continued to meet the inclusion criteria until one of the following endpoints has been reached: 1) CR or response able to achieve successful downstaging within the Milan criteria; 2) technical impossibility to embolize the residual tumor, for example, in a tumor only supplied by extrahepatic collateral arteries; 3) development of contraindications to DSM-TACE; 4) PD after each of two consecutive DSM-TACE treatments; and 5) worsening of liver function up to Child-Pugh stage C.

### Outcomes

The primary efficacy endpoint was the time-to-event analysis (time-to-downstaging), which examined the efficacy of DSM-TACE as a downstaging therapy in a patient with stage B HCC and a Child-Pugh score of 8/9. Patients’ data were censored at the end of the follow-up (October 31, 2020), at the time of study withdrawal, at the time HCC stage progressed, or if death had occurred. The secondary efficacy endpoints included the radiological response to treatment, the overall survival, the progression-free survival, and the proportion of patients successfully downstaged. The overall survival was calculated as the time from the study inclusion date until death or the last follow-up. The progression-free survival was measured from the study inclusion date to disease progression.

The primary safety endpoint was the incidence of serious adverse events, in accordance with the classification set out in the next paragraph. The secondary safety endpoints were the incidence and severity of adverse events, including liver function parameters and laboratory abnormalities.

### Definitions

Technical success was defined as the ability to deliver the full planned dose of doxorubicin and to obtain stop flow (Basile et al., 2012). Treatment response was assessed using mRECIST guidelines (Lencioni and Llovet, 2010). Complete response was defined as the disappearance of any intratumoral arterial enhancement in all target lesions. Partial response was defined as at least a 30% decrease in the sum of the diameters of viable (contrast-enhancing) target lesions. Progressive disease was defined as an increase of at least 20% in the sum of the diameters of the viable (enhancing) target lesions, and stable disease included all cases that did not qualify as either partial response or progressive disease. Patients developing new lesions, vascular invasion, and/or metastases were categorized as having PD. As previously reported (Lammer et al., 2010; Zhang et al., 2018), disease control was defined and calculated as CR + PR + SD. Responders referred to objective response, namely the sum of patients who experienced CR or PR. Non-responders referred to the sum of patients who had stable disease or progressive disease. The initial response was defined as the radiological response after the first DSM-TACE. The best response was defined as the best radiological response across repeated DSM-TACE sessions. Patients who achieved an objective response after the first treatment or after the following ones were considered as initial or best responders, respectively. Sustained response duration was defined as the time between the date when CR, PR, or stable disease was achieved and the date progressive disease occurred.

All adverse events were graded using the National Cancer Institute Common Terminology Criteria for adverse events, version 4.0 (National Institute of Cancer, 2010), except for clinical complications associated with chemoembolization recorded using the CIRSE Classification System for Complications (Filippiadis et al., 2017). In reference to the CTCAE, toxicity was further graded using binary variables (mild: grades 1–2; serious: grades 3–4), adapted, and modified from (Kang et al., 2020).

### Statistical Analysis

Data were maintained in an Excel spreadsheet (Microsoft Inc., Redmond, Wash), and the statistical analyses were performed using SPSS software (SPSS, version 22 for Windows; SPSS Inc., Chicago IL, United States) and R/R Studio software. The analysis of efficacy was based on the Modified Intention-To-Treat population, defined as all randomized patients who received at least one chemoembolization; this also defined the safety population. The Kolmogorov-Smirnov test and Shapiro-Wilk test were used to verify the normality assumption of data. Categorical data are presented as frequency (percentage value). Continuous normally distributed data are presented as mean ± standard deviation. Continuous not normally distributed data are presented as median (interquartile range: 25th and 75th percentiles—IQR). The unpaired Student t-test was used to assess statistical differences for continuous normally distributed data, while categorical and continuous not normally distributed data were assessed using the Chi-squared test and the Mann-Whitney test, respectively. Kaplan-Meier survival analysis was performed to assess time-dependent outcomes, and comparisons were made with the log-rank test. The independence between censored data and the tested events was assessed by clinical evaluation and telephone contacts in the cases of withdrawal. Hence, the assumption of independent censoring was met, avoiding bias regarding the observed time-dependent data. Among all survivors (with and without drop-out from the transplant list), follow-up ended on October 31, 2020. Univariate and multivariate analyses, using Cox proportional hazards and logistic regression models, were performed to identify individual predictors (patient/lesion characteristics) associated with successful downstaging while controlling for all other predictors in the model. A *p*-value of <0.05 was considered statistically significant for the aforementioned tests.

## Results

### Patient and Pathology Data

Between January 2015 and October 2020, 50 consecutive patients with intermediate-stage hepatocellular carcinoma and a Child-Pugh score of 8/9, who had undergone DSM-TACE as the first-line treatment, were eligible for the study. All patients received at least one chemoembolization treatment, meeting the criteria to be included in the Modified Intention-To-Treat (MITT) population. No patients were lost to follow-up. The mean age was 44.1 years and 80% of the patients were male. Among liver comorbidities, 12% of the patients had the hepatitis B virus, 40% the hepatitis C virus, 8% non-alcoholic fatty liver disease, and 44% alcoholic liver disease. The median alpha-fetoprotein and carbohydrate antigen 19–9 levels at the time of listing were 447 ng/ml and 11.3 U/ml, respectively. All patients were affected by cirrhosis; Child-Pugh score was B8 (84%) or B9 (16%). A total of 24% of the patients had encephalopathy, while none had ascites. The median values of neutrophil-to-lymphocyte ratio and lymphocyte-to-monocyte ratio were 3.8 and 8.4, respectively. In total, 36% of the patients had one tumor nodule, 32% of the patients had two tumor nodules, and the other 32% had three tumor nodules; the median maximum tumor size was 4.1 cm (3.3–5.1 cm). A total of 22 patients (44%) had bilobar disease, while 28 patients (56%) had capsulated tumors.

Demographics and tumor data of the study population are reported in Table 1.

**TABLE 1:** Population data.

Variables	All patients (n=50)
Age (years)	44.1 (±14.8)
Sex (M/F)	40 (80%) / 10 (20%)
Hepatitis B virus	6 (12%)
Hepatitis C virus	20 (40%)
Non-alcoholic fatty liver disease	4 (8%)
Alcoholic liver disease	22 (44%)
α-Fetoprotein (ng/ml)	447 (25-9670.9)
Carbohydrate antigen 19-9 (U/ml)	11.3 (1.7-29.8)
γ-Glutamyltransferase (U/L)	98 (12-196)
Alkaline phosphatase (U/L)	50 (22-86)
Aspartate transaminase (U/L)	33 (20-63)
Alanine transaminase (U/L)	45 (36-80)
Albumin (g/L)	28 (26-37)
Total bilirubin (mg/dL)	1.2 (0.8-1.5)
Prothrombin time (seconds prolonged)	5 (4-9)
Ascites, no/yes	54 (100%) / 0 (0%)
Encephalopathy, no/yes	38 (76%) / 12 (24%)
Child-Pugh score, B9/B8	8 (16%) / 42 (84%)
Cirrhosis, no/yes	0 (0%) / 50 (100%)
Platelet count (No. x10^3^/μL)	99 (85-349)
Creatinine (mg/dL)	1.2 (0.6-1.4)
Hemoglobin (g/dl)	13.5 (12.1-14.9)
White blood cell count (per μL)	4011 (4001-4222)
Neutrophil count (per μL)	3012 (2989-3189)
Lymphocyte count (per μL)	812 (614-902)
Monocyte count (per μL)	108 (80-214)
Neutrophil-to-lymphocyte ratio (NLR)	3.8 (3.3-8.0)
Lymphocyte-to-monocyte ratio (LMR)	8.4 (1-11.2)
Number of Tumors, 1/2/3	18 (36%) / 16 (32%) / 16 (32%)
Maximum tumour size (cm)	4.1 (3.3-5.1)
Bilobar disease, no/yes	28 (56%) / 22 (44%)
Capsule, absent/present	22 (44%) / 28 (56%)

### Procedure Data

A total of 142 DSM-TACEs were performed, with a mean number of 2.84 procedures per patient. The chemoembolization pattern was selective in 133 procedures (93.7%) and lobar in nine procedures (6.3%); no procedure was performed with the catheter placed in the common hepatic artery.

Procedure data are reported in Table 2.

**TABLE 2:** Procedure and outcomes data.

Variables		All patients (n=50)
Total number of DSM-TACEs		142
Mean number of DSM-TACEs per patient		2.84
Mean follow-up (months)		20.8
Technical success, no/yes		0 (0%) / 142 (100%)
Chemoembolization pattern		
	Selective/Superselective	133 (93.7%)
	Lobar	9 (6.3%)
	Global	0
Tumour response to first DSM-TACE (No.)		50
	CR	4 (8%)
	PR	12 (24%)
	SD	26 (52%)
	PD	8 (16%)
	Non-responders (SD+PD)	34 (68%)
	Responders or OR (CR+PR)	16 (32%)
	DC (CR+PR+SD)	42 (84%)
Tumour response to second DSM-TACE (No.)		38
	CR	2 (5.3%)
	PR	6 (15.8%)
	SD	16 (42.1%)
	PD	14 (36.8%)
	Non-responders (SD+PD)	30 (78.9%)
	Responders or OR (CR+PR)	8 (21%)
	DC (CR+PR+SD)	24 (63.1%)
Tumour response to third DSM-TACE (No.)		34
	CR	0 (0%)
	PR	14 (41.2%)
	SD	10 (29.4%)
	PD	10 (29.4%)
	Non-responders (SD+PD)	20 (58.8%)
	Responders or OR (CR+PR)	14 (41.2%)
	DC (CR+PR+SD)	24 (70.6%)
Tumour response to fourth DSM-TACE (No.)		20
	CR	0 (0%)
	PR	0 (0%)
	SD	6 (30%)
	PD	14 (70%)
	Non-responders (SD+PD)	20 (100%)
	Responders or OR (CR+PR)	0 (0%)
	DC (CR+PR+SD)	6 (30%)
Best Response (No.)		50
	CR	6 (12%)
	PR	28 (56%)
	SD	12 (24%)
	PD	4 (8%)
	Non-responders (SD+PD)	16 (32%)
	Responders or OR (CR+PR)	34 (68%)
	DC (CR+PR+SD)	46 (92%)
Sustained Response Duration (SRD), <6 months/≥6 months		24 (48%) / 26 (52%)
Time-to-downstaging (months)		19.2 (±9.3)
Event, censoring/death		22 (44%) / 28 (56%)
Event, censoring/downstaging		44 (88%) / 6 (12%)
Post-procedural clinical complications (CIRSE class.), absent/present		34 (68%) / 16 (32%)
	Grade 1	14 (28%)
	Grade 2	0 (0%)
	Grade 3	2 (4%)
Adverse Events (CTCAE), absent/present		28 (56%) / 22 (44%)
	Grade 1	12 (24%)
	Grade 2	8 (16%)
	Grade 3	2 (4%)
	Grade 4	0 (0%)
	Serious Adverse Events	2 (4%)

### Efficacy Outcomes

Technical success was achieved in 142 procedures (100%). The average follow-up was 20.8 months. After the first DSM-TACE, CR was achieved in four out of 50 patients (8%), PR in 12 (24%), SD in 26 (52%), and PD in eight (16%), with 34 (68%) non-responders, 16 (32%) responders, and disease control achieved after 42 (84%) procedures. A second DSM-TACE was performed in 38 cases, after which CR was achieved in two patients (5.3%), PR in six (15.8%), SD in 16 (42.1%), and PD in 14 (36.8%), with 30 (78.9%) non-responders, 8 (21%) responders, and disease control achieved after 24 (63.1%) procedures. A third DSM-TACE was performed in 34 cases, after which no CR was achieved, PR was achieved in 14 patients (41.2%), SD in 10 (29.4%), and PD in 10 (29.4%), with 20 (58.8%) non-responders, 14 (41.2%) responders, and disease control achieved after 24 (70.6%) procedures. A fourth DSM-TACE was performed in 20 cases, after which no CR and PR were achieved, SD was achieved in six patients (30%) and PD in 14 (70%), with 20 (100%) non-responders, 0 (0%) responders, and disease control achieved after six (30%) procedures. Considering the best response across repeated DSM-TACE sessions for each patient, CR was achieved in six out of 54 patients (12%), PR in 28 (56%), SD in 12 (24%), and PD in four (8%), with 16 (32%) non-responders, 34 (68%) responders, and disease control achieved in 46 patients (92%). Twenty-six (52%) patients achieved a sustained response duration (SRD) of six months or more; the rest of the patients (48%) achieved an SRD of less than six months. The mean time-to-downstaging was 19.2 (±9.3) months, with six patients (12%) successfully downstaged. The cumulative rates of patients successfully downstaged were about 8% (±0.04) at six months, 12% (±0.05) at 12 months, and 12% (±0.05) at 24 months. Death occurred in 28 cases (56%) along the follow-up period. OS was about 100% (±0.00) at six months, 81.8% (±0.06) at 12 months, and 50% (±0.07) at 24 months. For patients with SRD of more than six months, the median (CI) OS was 35 (35-NA) months, better than that of patients with SRD of less than six months (median OS, 16.5 [15–23] months) (*p* = 0.0003, calculated by mean of Log-Rank test). Progression-free survival was about 66% (±0.07) at six months, 37% (±0.08) at 12 months, and 16% (±0.06) at 24 months with only two residual patients at risk.

**FIGURE 1:**
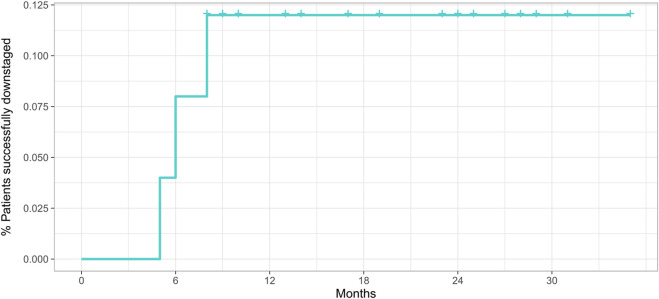
Cumulative incidence of patients successfully downstaged.

**FIGURE 2:**
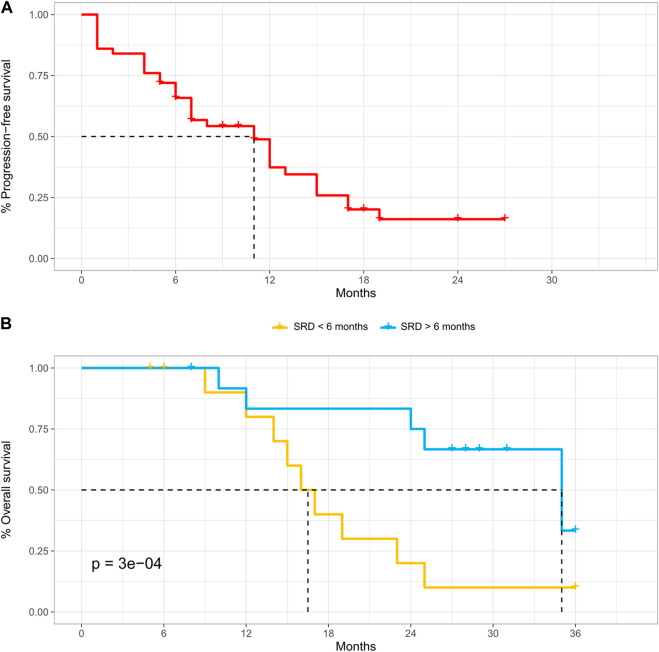
**(A–B)** Time-to-event analysis (listing to progression) **(A)** and time-to-event analysis (listing to death), according to sustained response duration (SRD) **(B)**.

The efficacy outcomes are shown in Table 2, Table 3, Figure 1, and Figure 2.

**TABLE 3:** Time-to-event outcomes as indicated in the related survival plots (Figures 1, 2).

Cumulative Rates	At 6 months	At 12 months	At 24 months
	rate (±SE)—numbers at risk	rate (±SE)—numbers at risk	rate (±SE)—numbers at risk
Patients successfully downstaged	8% (±0.04)—48	12% (±0.05)—34	12% (±0.05)—22
Progression-free Survival (PFS)	66% (±0.07)—35	37% (±0.08)—17	16% (±0.06)—2
OS according to SRD < 6 m	100% (±0.00)—22	80% (±0.09)—18	20% (±0.09)—4
OS according to SRD > 6 m	100% (±0.00)—26	83% (±0.08)—22	75% (±0.09)—20

Details of the predictors of successful downstaging are listed in Table 4. Based upon the intention to treat on both univariate and multivariate analyses, number of tumor nodules equal to one (HR, 1.4; 95% CI, 1.0–1.7; P 0.02), presence of tumor capsule (HR, 1.4; 95% CI, 1.0–1.7; P 0.02), objective response as the best response across repeated DSM-TACE sessions for each patient (HR, 1.5; 95% CI, 1.1–2.0; P 0.01), objective response as the initial response (HR, 1.5; 95% CI, 1.2–2.0; P 0.01), SRD of six months or more (HR, 1.6; 95% CI, 1.2–2.2; *p* < 0.01), and lymphocyte-to-monocyte ratio ≥ 4/neutrophil-to-lymphocyte ratio < 7.2 (HR, 1.7; 95% CI, 1.5–2.4; *p* < 0.01) were found to be the independent prognostic factors for successful downstaging.

**TABLE 4:** Factors predicting successful downstaging.

Variable	Univariate	Multivariate
	HR (95% CI)–P value	HR (95% CI) – *p* value
Age	1.1 (0.8–1.3)–0.80	NA
Age ≥60 years	0.9 (0.7–1.1)–0.78	NA
Sex (Male)	0.9 (0.7–1.3)–0.81	NA
Hepatitis B virus	1.1 (0.9–1.5)–0.79	NA
Hepatitis C virus	0.9 (0.8–1.1)–0.73	NA
Non-alcoholic fatty liver disease	1.2 (0.8–1.3)–0.61	NA
Alcoholic liver disease	0.9 (0.8–1.2)–0.76	NA
α-Fetoprotein (ng/ml) > 300	0.9 (0.7–1.3)–0.80	NA
Aspartate transaminase (>40 U/L vs ≤40)	1.1 (0.9–1.3)–0.81	NA
Alanine transaminase (>40 U/L vs ≤40)	1.1 (1.0–1.3)–0.79	NA
Tumour nodules No. (1 vs ≥2)	1.4 (1.0–1.8)–0.02	1.4 (1.0–1.7)–0.02
Capsule (present vs absent)	1.4 (1.0–1.8)–0.02	1.4 (1.0–1.7)–0.02
Objective response as the best response	1.5 (1.1–1.9)–0.01	1.5 (1.1–2.0)–0.01
Objective response as the initial response	1.5 (1.2–2.0)–0.01	1.5 (1.2–2.0)–0.01
SRD (≥6 months vs <6 months)	1.6 (1.3–2.1)–<0.01	1.6 (1.2–2.2)–<0.01
LMR / NLR (≥ 4 / < 7.2 vs. < 4 / ≥ 7.2)	1.6 (1.4–2.3)–<0.01	1.7 (1.5–2.4)–<0.01

### Safety Outcomes

According to the CIRSE Classification System for Complications, 16 patients (32%) experienced postprocedural clinical complications associated with chemoembolization. Apart from two treatment-related grade 3 events (non-surgical cholecystitis), only grade 1 events occurred (14 cases, 28%). These were pain responses to analgesics (10 DSM-TACEs, 20%), post-embolization syndrome (2 DSM-TACEs, 4%), transient nausea (1 DSM-TACEs, 2%), and vomiting (1 DSM-TACEs, 2%). The aforementioned adverse events were transient and easily solved with standard analgesic or antiemetic medication during interventions.

According to the CTCAE classification, 22 patients (44%) experienced adverse events after chemoembolization. Grade 1 events were observed in 12 out of 54 patients (24%), grade 2 events in eight (16%), and grade 3 events in two (4%); no grade 4 adverse events were observed. Hence, only two (4%) serious adverse events occurred, namely, grade 3 or 4 adverse events according to the Common Terminology Criteria for adverse events.

Details are given in Table 2.

## Discussion

Current guidelines consider TACE the standard of care for patients with intermediate-stage HCC who are not LT candidates, according to the BCLC staging system (EASL guidelines, 2018; ESMO guidelines, 2018) (Galle et al., 2018; Vogel et al., 2018). Since two randomized controlled trials demonstrated an improvement of OS with TACE compared to best supportive care, it has been a mainstay of treatment for unresectable HCCs (Llovet et al., 2002; Lo et al., 2002), identifying treatment response as an independent predictor of survival (Llovet and Bruix, 2003). However, it is not a risk-free method and so to reduce the systemic concentration of the cytotoxic drug, maximizing instead the intratumoral one, and to obtain a calibrated ischemia of the tumor vessel, alternative procedures have been studied such as DEB-TACE and DSM-TACE (Varela et al., 2007; Gross and Albrecht, 2020). Temporary ischemia should give a better safety profile, making DSM-TACE a valid alternative for multifocal or advanced disease with liver disfunction when highly selective TACE is not feasible (Gross and Albrecht, 2020). Most of the transarterial approaches may be repeated several times when an incomplete response is achieved or in the case of tumor recurrence with an excellent safety profile, in a high volume center (Sieghart et al., 2013). Moreover, intermediate-stage HCC (B-BCLC) represents a highly heterogeneous scenario which would require more reasoned sub-classification (Bolondi et al., 2012). Indeed, this population includes patients with multinodular, not severely compromised liver function (Child-Pugh A and B) and ECOG PS 0; it is clear that within this definition there can be patients with very different clinical, morphological, and prognostic features. Particularly, as per current guidelines, patients with bulky disease, multinodular spread, or compromised liver function may not be optimal TACE candidates and other LRT should be evaluated (Galle et al., 2018). In patients with intermediate-stage HCC and a Child-Pugh score of 8 or 9, life expectation is negatively influenced by cirrhotic liver dysfunction, rather than by the tumor growth itself (Bolondi et al., 2012). Furthermore, it is widely accepted that patients outside the Milan criteria can be considered for liver transplant after successful downstaging to within the Milan criteria, within specified protocols (Galle et al., 2018). Response to downstage therapies has a major effect on the rate of drop-outs, on the rate of post-transplantation tumor recurrences (Cucchetti et al., 2011; Yao et al., 2015), and is linked to the presence of good prognosis histology, (i.e. the absence of microvascular invasion and satellites, low-grade tumor), similar to patients that met the Milan criteria already at the diagnosis. Therefore, the downstage response plays an important role in the prediction of tumor aggressiveness (Mazzaferro et al., 2011; Yao et al., 2015). Additionally, down-staged tumors, initially exceeding the Milan Criteria, can achieve post-transplant five-year survival and HCC recurrence-free probability just like patients that ab-initio met the Milan criteria (Yao et al., 2015). To give further insight into this unmet medical need, this study aimed to evaluate the safety and efficacy of DSM-TACE in the downstaging of intermediate-stage HCC in patients with a Child-Pugh score of 8 or 9.

With regard to tumor burden, in our analysis, the percentage of patients with one, two, or three tumors were homogeneous, with a maximum tumor size of 5.1 cm at the third quartile and bilobar disease in 44% of patients. In other prospective trials, tumors were smaller with a median tumor size of 2.6 cm and 20.9% had bilobar disease (Golfieri et al., 2014), while in the study of Gross et al. tumor burden was slightly higher. The mean time-to-downstaging, the primary endpoint of this study, was 19.2 (±9.3) months, with 12% of patients successfully downstaged. The cumulative rates of patients successfully downstaged were about 8% (±0.04) at six months, 12% (±0.05) at 12 months, and 12% (±0.05) at 24 months. OS was about 100% at six months, 81.8% at 12 months, and 50% at 24 months. To the best of our knowledge, this is the first study exploring the role of SRD as a prognostic factor after DSM-TACE. For patients with SRD of more than six months, the median OS was 35 months, better than that of patients with SRD of less than six months (median OS, 16.5 months). Based upon the intention to treat on both univariate and multivariate analyses, the number of tumor nodules equal to one, presence of tumor capsule, objective response as the best response across repeated DSM-TACE sessions for each patient, objective response as the initial response, SRD of six months or more, and lymphocyte-to-monocyte ratio ≥4/neutrophil-to-lymphocyte ratio <7.2 were found to be the independent prognostic factors for successful downstaging. Zhang et al. reported similar data using downstaging cTACE concluding that tumor burden (tumor number, tumor size) and tumor structure (tumor capsule) have a pivotal role in determining overall success rates of procedures (Zhang et al., 2018). Our analysis also shows the role of achieving an objective response after the first treatment, an SRD of six months or more and other parameters such as LMR≥ 4 and NLT <7.2. Particularly these data confirm what was observed using cTACE (Zhang et al., 2018), namely that the maintenance of response, rather than achieving the radiological objective response itself, may be more clinically important for long-term outcomes because they are more related to histological tumor necrosis. Furthermore, in the era of immunotherapy, the immune response stimulation induced by LRT has a non-negligible role to an extent that the efficacy outcomes could be determined not only by LRT but also by the immune response induced by LRT itself. Besides, PFS was about 66% at six months, 37% at 12 months, and 16% at 24 months with only two residual patients at risk. A high objective response rate was observed (68% of patients showed CR or PR), reaching a disease control of 92% and a sustained response duration (SRD) of six months or more of 52%. Gross et al. reported an objective response rate assessed with mRECIST for B-BCLC stage of 49% (7/37 patients had a B Child-Pugh class).

The safety of DSM-TACE in this study is comparable with other published investigations in this field. According to the CTCAE classification, 44% of the patients experienced adverse events after chemoembolization. The most frequent adverse events, all of these transient and responsive to medical treatment, was pain (20%), post-embolization syndrome (4%), nausea, and vomiting (2%). Hence, only two serious adverse events occurred (non-surgical cholecystitis). For DSM-TACE in intermediate-stage HCC patients, Gross et al. reported abdominal pain in 23%, nausea in 11%, vomiting in 3%, and post-embolization syndrome (4%) (Gross and Albrecht, 2020). Interestingly, adverse events reported for cTACE included fever in 57.8%, abdominal pain in 42.5%, nausea in 32.4%, vomiting in 34.2%, and post-embolization syndrome in 47.7%, and for DEB-TACE in the PRECISION V trial, 20.4% of patients had 6 major complications and 84% of patients had post-embolization syndrome after the first treatment cycle (Lammer et al., 2010; Brown et al., 2016; Lencioni et al., 2016; Gruber-Rouh et al., 2018).

Limitations of this study include the fact that the research was based on a retrospective, single-center, and non-randomized design, the small study population and the lack of literature data in this field needed to evaluate the congruence and the consistency of the data presented. Thus, randomized-controlled clinical trials should be performed to validate our results in the downstaging of intermediate-stage hepatocellular carcinoma in patients with a Child-Pugh score of 8-9.

## Conclusion

To the best of our knowledge, no observational studies have so far investigated the efficacy and safety profile of DSM-TACE in the downstaging of intermediate-stage hepatocellular carcinoma in patients with a Child-Pugh score of 8-9.

Hence, the results of the current investigation demonstrate that DSM-TACE is effective and safe in intermediate-stage HCC, achieving an interesting downstaging rate. Such data were observed in the population subset with a Child-Pugh score of 8 or 9, in which life expectancy may be dominated by cirrhotic liver dysfunction, rather than by the tumor progression itself, so that the achievement of a balance between the safety and efficacy profile of TACE treatment is crucial.

Larger, randomized controlled trials are necessary to confirm these preliminary data.

## Data Availability

The raw data supporting the conclusions of this article will be made available by the authors, without undue reservation.

## Abbreviations

AASLD, American Association for the Study of Liver Diseases; AEs, adverse events; BCLC, Barcelona-Clinic Liver Cancer; CT, computed tomography; cTACE, conventional transarterial chemoembolization; DC, disease control; DEB, drug-eluting beads; DSM, degradable starch microspheres; ESMO, European Society for Medical Oncology; HCC, hepatocellular carcinoma; HIFs, hypoxia-inducible factors; LMR, lymphocyte-to-monocyte ratio; LRTs, locoregional treatments; LT, Liver transplantation; MELD, model for end-stage liver disease; MITT, modified intention-to-treat; NLR, neutrophil-to-lymphocyte ratio; OR, objective response; OS, overall survival; PFS, progression-free survival; RFA, radiofrequency ablation; SAEs, serious adverse events; SRD, sustained response duration; TACE, transarterial chemoembolization; TFV, tumor feeding vessel; VEGF, vascular endothelial growth factor; WL, waiting list
